# Programmed Death-1 Expression on Epstein Barr Virus Specific CD8+ T Cells Varies by Stage of Infection, Epitope Specificity, and T-Cell Receptor Usage

**DOI:** 10.1371/journal.pone.0012926

**Published:** 2010-09-23

**Authors:** Thomas C. Greenough, Shalyn C. Campellone, Robin Brody, Surbhi Jain, Victor Sanchez-Merino, Mohan Somasundaran, Katherine Luzuriaga

**Affiliations:** 1 Pediatrics, University of Massachusetts Medical School, Worcester, Massachusetts, United States of America; 2 Molecular Medicine, University of Massachusetts Medical School, Worcester, Massachusetts, United States of America; 3 Medarex, Inc., Bloomsbury, New Jersey, United States of America; New York University, United States of America

## Abstract

**Background:**

Programmed Death-1 (PD-1) is an inhibitory member of the CD28 family of molecules expressed on CD8+ T cells in response to antigenic stimulation. To better understand the role of PD-1 in antiviral immunity we examined the expression of PD-1 on Epstein-Barr virus (EBV) epitope-specific CD8+ T cells during acute infectious mononucleosis (AIM) and convalescence.

**Methodology/Principal Findings:**

Using flow cytometry, we observed higher frequencies of EBV-specific CD8+ T cells and higher intensity of PD-1 expression on EBV-specific CD8+ T cells during AIM than during convalescence. PD-1 expression during AIM directly correlated with viral load and with the subsequent degree of CD8+ T cell contraction in convalescence. Consistent differences in PD-1 expression were observed between CD8+ T cells with specificity for two different EBV lytic antigen epitopes. Similar differences were observed in the degree to which PD-1 was upregulated on these epitope-specific CD8+ T cells following peptide stimulation *in vitro*. EBV epitope-specific CD8+ T cell proliferative responses to peptide stimulation were diminished during AIM regardless of PD-1 expression and were unaffected by blocking PD-1 interactions with PD-L1. Significant variability in PD-1 expression was observed on EBV epitope-specific CD8+ T cell subsets defined by V-beta usage.

**Conclusions/Significance:**

These observations suggest that PD-1 expression is not only dependent on the degree of antigen presentation, but also on undefined characteristics of the responding cell that segregate with epitope specificity and V-beta usage.

## Introduction

Programmed death 1 (PD-1) and its ligands, PD-L1 and PD-L2, are members of the CD28 and B7 families of proteins that participate in regulating the balance between T cell activation and tolerance [1], [2], [3], [4]. The interaction of PD-1 with its ligands delivers an inhibitory secondary signal to T cells that results in the downregulation of T cell receptor signaling and the suppression of T cell effector functions, including T cell proliferation and cytokine secretion. This downregulation of T cell responses is thought to be important in the generation of central and peripheral tolerance and in limiting immune pathology in chronic infections. In the context of acute viral infections, PD-1 may also play a role in the contraction of the immune response following acute infection [5].

High levels of PD-1 expression on virus-specific CD8+ T cells during chronic human (human immunodeficiency virus [HIV], hepatitis B virus [HBV]), monkey (simian immunodeficiency virus [SIV]), and murine (lymphocytic choriomeningitis virus [LCMV]) infections have been associated with T cell dysfunction and failure to control viral replication [6], [7], [8], [9], [10], [11], [12], [13], [14], [15], [16], [17], [18], [19], [20]. However, Kasprowicz and colleagues have recently reported high levels of PD-1 expression on peripheral blood hepatitis C virus (HCV)-specific CD8+ T cells of infected individuals who clear infection, raising the question of whether PD-1 expression alone is sufficient to confer T cell dysfunction [10]. In non-persistent viral infections, PD-1 expression is downregulated with the resolution of acute infection and viral clearance. Salisch and colleagues have recently reported that the upregulation and maintenance of high PD-1 expression levels on antigen-specific CD8+ T cells is dependent on continued activation through the T cell receptor in lentiviral infections [16].

Epstein Barr virus (EBV) infects over 95% of the world's population but is only rarely associated with disease despite persistent viral replication in chronically infected individuals [21]. Primary EBV infection in adolescence and young adulthood is often symptomatic, with a syndrome characterized by fever, malaise, pharyngitis and lymphadenopathy (acute infectious mononucleosis, AIM; reviewed in [22]). Vigorous EBV-specific CD4+ and CD8+ T cell responses are expanded early in AIM [23], [24], [25]; several lines of evidence suggest that these cellular immune responses are important for limiting primary EBV infection and controlling chronic infection such that diseases associated with EBV are relatively rare.

Human EBV infection has served as a useful model in efforts to characterize the lineage relationship between effector and memory responses and the factors that influence evolution of antigen-specific CD8+ T cell responses into the memory CD8+ T cell repertoire. Current models of EBV viral immunology link the phenotypes of EBV-specific CD8+ T cells detected in the circulation with the levels of viral antigen expression and/or the qualitative differences in their presentation [24], [26], [27], [28], [29], [30]. Several investigators have reported high levels of PD-1 expression on EBV-specific CD8+ T cells during chronic infection [8], [13]. Sauce and colleagues have reported that EBV-specific CD8+ T cells culled from the circulation during convalescence transiently express PD-1 at high levels concurrent with gradually increasing IL-7R (CD127) expression [31]. We studied PD-1 expression levels on EBV-specific CD8+ T cells during AIM, convalescence and chronic infection to better define its relationship with viral replication, the contraction of the immune response, and CD8+ T cell functional properties.

## Methods

### Ethics Statement

The Institutional Review Board of the University of Massachusetts Medical School approved these studies, and all participants provided written informed consent.

### Study Population

Students at the University of Massachusetts, Amherst campus were invited to participate in this study when they presented to the infirmary with symptoms consistent with acute infectious mononucleosis (AIM) and a positive monospot (heterophile antibody) assay. All were confirmed to have acute EBV infection by the detection of IgM antibodies to the EBV Viral Capsid Antigen. Blood samples were collected at baseline, weekly for six weeks, followed by long-term follow-up at six months and one year. Individuals with EBV-specific CD8+ T cells detected using HLA-A*0201 based, peptide-MHC Class I (pMHCI) tetramers (described below) were selected for more detailed studies.

### Flow Cytometry

Whole blood samples were screened for EBV-specific CD8+ T cells that reacted with APC-conjugated pMHCI tetramers including HLA-A*0201 based reagents: A2-BMLF-1 (epitope: GLCTLVAML), A2-BRLF-1 (epitope: YVLDHLIVV), A2-EBNA-3c (epitope: LLDFVRFMGV), and the HLA-B*0702-based reagent: B7-EBNA-3a (epitope: RPPIFIRRL). All pMHCI tetramer complexes were made in the Tetramer Core facility at UMMS as previously described [32].

PBMC samples were studied using a PE-conjugated antibody specific for PD-1 (Medarex). Antibodies from BD-Biosciences (San Jose, CA) were used to detect: CD27 (Alexa700), CD28 (PE-Cy7), CD127 (FITC or PE), HLA-DR (FITC or PerCP). A panel of FITC-conjugated antibodies specific for different V-beta subsets (Immunotech/Beckman Coulter) was used in a four-color assay that also included CD8 (PerCP), PD-1 (Medarex; PE), and Tetramer (APC). For multi-parameter flow assays, a “dump channel” of cells positive for CD14 and CD19 (APC-Cy7) was used to improve specificity, and live/dead blue reagent (Invitrogen) was used to exclude dead cells. Unstained and “fluorescence minus one” samples were included as controls in all experiments to optimize specificity during analyses.

Assays involving≤four fluorochromes utilized FACScan or FACSCalibur instruments (BD Biosciences), with CellQuest software (BD Biosciences). For experiments involving>four fluorochromes, we used a LSRII instrument (BD Biosciences). Multiparameter flow cytometry data were analyzed using FlowJo software (Treestar, Ashland OR) and programs generously provided by Mario Roederer (PESTLE and SPICE version 4.2).

A complete blood count with differential was obtained at each study visit. The absolute lymphocyte count was calculated by multiplying the white blood cell count (#/ml) by the lymphocyte percentage. The absolute CD8+ T cell count was calculated by multiplying the absolute lymphocyte count by the CD8+ percentage. The absolute counts of virus-specific CD8+ T cells were calculated by multiplying the absolute CD8+ T cell count by the percentages of epitope-specific CD8+ T cells.

The PD-1 median fluorescence index (MFI) values on virus-specific CD8+ T cells were expressed as normalized values: the ratio between the PD-1 median fluorescence intensity and the cutoff between PD-1 positive and negative CD8+ T cells.

### Peptide Stimulation

Cryopreserved PBMC were thawed into RPMI 1640, containing 10% heat inactivated human AB sera, 2mM L-glutamine, 100 U/ml gentamicin (R10 medium; all materials from Gibco), with 2 mg/ml DNase (Sigma) and pelleted. Cells were re-suspended at 10 million/ml, incubated with CFSE (Invitrogen) at a final concentration of 0.5µM for 10 minutes at 37°C, 5% CO2 and 95% humidity. Cells were cultured at 1.5 to 3 million cells/ml in R10 medium containing co-stimulatory antibodies, CD49d and CD28 (final concentration of 1ug/ml; both from BD Pharmingen), the EBV peptide of interest (final concentration 10 µg/ml), no peptide, or Candida antigen. Cells in each assay condition were incubated at 150,000 to 300,000 cells per well in 96-well microtiter plates, in replicates of 10. After 6 days, they were harvested, pooled, treated with 20 mM EDTA for 15 minutes, and washed with 1% BSA/PBS in preparation for antibody staining. Cells were then stained with relevant APC-conjugated pMHCI tetramers, PD-1 (PE) (Medarex), and CD8 (PerCP; BD Biosciences). Anti-PD-L1 antibody (EBioscience, # 16-5983), when included in blocking assays, was used at a concentration of 25 µg/ml and was added simultaneously with peptide.

### EBV DNA Quantitation in B Cells

B-cells were purified from whole blood using the RosetteSep human B-cell enrichment cocktail and manufacturer's protocol (StemCell Technologies, Vancouver BC, Canada). Cellular DNA was extracted using QIAGEN DNeasy mini spin columns (Valencia, CA). The Roche LightCycler EBV Quantitation Kit was used to quantify EBV DNA copy number. Cell counts in each sample were determined using a previously described PCR assay to quantify the copy number of the gene encoding CCR5 (two copies per diploid cell) [25].

### Statistics

GraphPad Prism version 5.0 for Mac OSX (GraphPad Software, La Jolla, CA) was used for all statistical analyses. The Wilcoxon Signed Rank test was used for paired comparisons. The Spearman rank test was used for analyses of correlations. To determine if V-beta subsets had significantly different expression of PD-1 than the overall Tetramer positive subpopulation we used the range defined by the mean +/− 3*SD of PD-1 expression on Tetramer positive cells.

## Results

### PD-1 Expression is Upregulated on EBV-specific CD8+ T Cells in AIM

CD8+ T cells undergo marked expansion during AIM, primarily due to the expansion of EBV epitope-specific CD8+ T cells (reviewed in [33]). Lytic epitope-specific CD8+ T cell responses are most highly expanded during acute EBV infection; in some individuals, CD8+ T cells specific for a single lytic epitope may represent more than 25% of the total circulating CD8+ T cells [24], [30], [33]. Latent epitope-specific CD8+ T cells are less highly expanded during AIM and convalescence.

**Figure 1:**
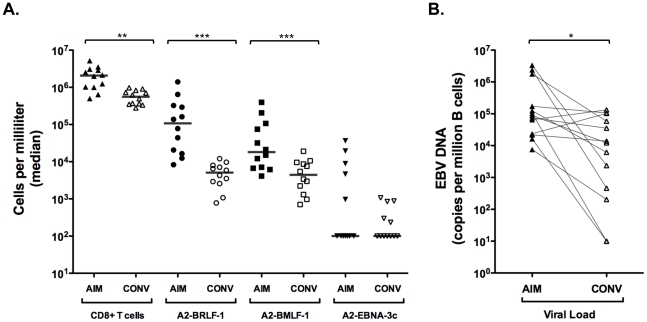
EBV-specific CD8+ T cells and viral load during AIM and convalescence. **A**. Absolute values (cells per ml) of CD8+ T cells, and three different EBV-specific subsets (lytic and latent antigen specific) measured in samples from individuals during AIM (within the first two weeks after presentation with symptoms) and convalescence (CONV; 6–12 months after presentation with symptoms). A2-BMLF-1 CD8+ T cells: specific for the HLA-A*0201 restricted epitope of BMLF-1, GLCTLVAML; A2-BRLF-1 CD8+ T cells: specific for the HLA-A*0201 restricted epitope of BRLF-1, YVLDHLIVV; A2-EBNA-3c CD8+ T cells: specific for the HLA-A*0201 restricted epitope of EBNA-3c, LLDFVRFMGV. When no EBV-specific CD8+ T cells were detected with these reagents, a value of 100 cells per milliliter was assigned (less than half the lowest detected value). **B**. Viral load (EBV copies per million B cells) during AIM and convalescence. Lines define paired results for each individual. All paired comparisons indicated by brackets differ significantly by the Wilcoxon signed rank test; * p<0.05, ** p<0.01, *** p<0.001.

We measured HLA A*0201-restricted, EBV-specific epitope-specific CD8+ T cells in peripheral blood samples obtained from 13 individuals during AIM (within the first two weeks after presentation with symptoms) and convalescence (6 to 12 months after presentation with symptoms; Figure 1A). CD8+ T cells were expanded in AIM and contracted (3.7-fold decrease in mean value) in convalescence. CD8+ T cells specific for an HLA-A*0201 restricted epitope in the immediate early lytic protein, BRLF-1, (YVLDHLIVV; hereafter abbreviated A2-BRLF-1 CD8+ T cells) were most highly expanded during AIM, and underwent the greatest degree of contraction (48.7-fold decrease in mean value) in convalescence. CD8+ T cells specific for an HLA A2-restricted epitope in the early lytic protein, BMLF-1, (GLCTLVAML; hereafter abbreviated A2-BMLF-1 CD8+ T cells) were also highly expanded during AIM, but underwent a smaller contraction (12.2-fold decrease in mean value) in convalescence. CD8+ T cells specific for an HLA A2-restricted epitope in the latent antigen, EBNA-3c, (LLDFVRFMGV; hereafter abbreviated A2-EBNA-3c CD8+T cells) were even less highly expanded (detected in only 5 of 13 individuals at each time point) and underwent a 17.6-fold decrease in mean value from AIM to convalescence. These changes in circulating EBV epitope-specific CD8+ T cell numbers coincided with a 17.5-fold decrease in mean peripheral blood EBV DNA copy number over the same time period (Figure 1B). They are consistent with previous reports showing a larger decline in A2-BRLF-1 CD8+ T cell frequencies than either A2-BMLF-1 or A2-EBNA-3c CD8+ T cells [21], [29].

**Figure 2:**
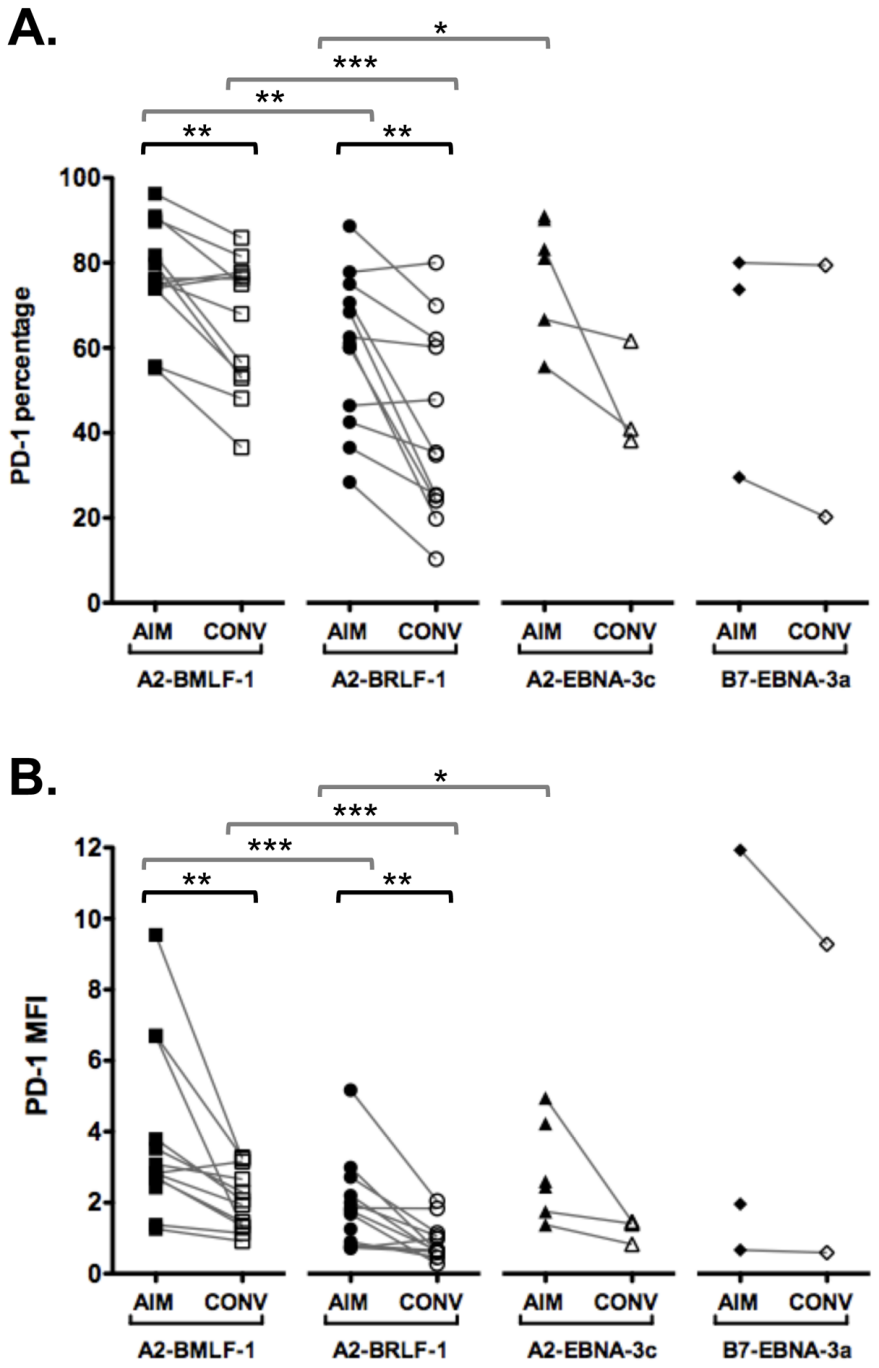
PD-1 expression on EBV-specific CD8+ T cells at AIM and convalescence. **A**. The percentages of EBV-specific CD8+ T cells that expressed PD-1 are shown. Two lytic (A2-BMLF-1 and A2-BRLF-1) and two latent (A2-EBNA-3c and B7-EBNA-3a CD8+ T cells) antigen specific CD8+ T cell subsets are shown. Lines define paired results for each individual. **B**. The median fluorescence index (MFI) of PD-1 expression for these EBV-specific CD8+ T cells is shown. All assays were performed with the PD-1 antibody conjugated to PE. The MFI was normalized as described in methods. Lines define paired results for each individual. All paired comparisons indicated by brackets differ significantly by the Wilcoxon signed rank test; * p<0.05, ** p<0.01, *** p<0.001.

We next examined PD-1 expression on EBV-specific CD8+ T cells in these individuals from AIM through convalescence. During AIM, 55.2 to 96.3% (median 76.3%) of A2-BMLF1-specific CD8+ T cells expressed PD-1 (Figure 2A). The frequencies of A2-BMLF1-specific CD8+ T cells expressing PD-1 remained high in convalescence (36.6 to 85.9%; median 71.5%), but were significantly lower than during AIM (Wilcoxon signed rank test, p<0.05). Similarly, high frequencies of PD-1+ A2-BRLF1-specific CD8+ T cells were detected during AIM (28.4 to 88.6%, median 61.0%), and decreased significantly in convalescence (10.3 to 80.0%, median 35.1%; Wilcoxon signed rank test, p<0.05). Similar trends were observed for two additional EBV epitope-specific responses (A2-EBNA-3c and B7-EBNA-3a CD8+ T cells, Figure 2A).

Higher intensity of PD-1 expression was also observed on EBV epitope-specific CD8+ T cells during AIM compared with convalescence (Figure 2B). The PD-1 median fluorescence index (MFI) values on A2-BMLF-1 (mean = 3.9) and A2-BRLF-1 (mean = 2.0) CD8+ T cells during AIM were significantly higher than during convalescence (mean = 2.1 for A2-BMLF-1 and 0.9 for A2-BRLF-1 CD8+ T cells); a similar trend was observed for A2-EBNA-3c CD8+ T cells.

**Figure 3:**
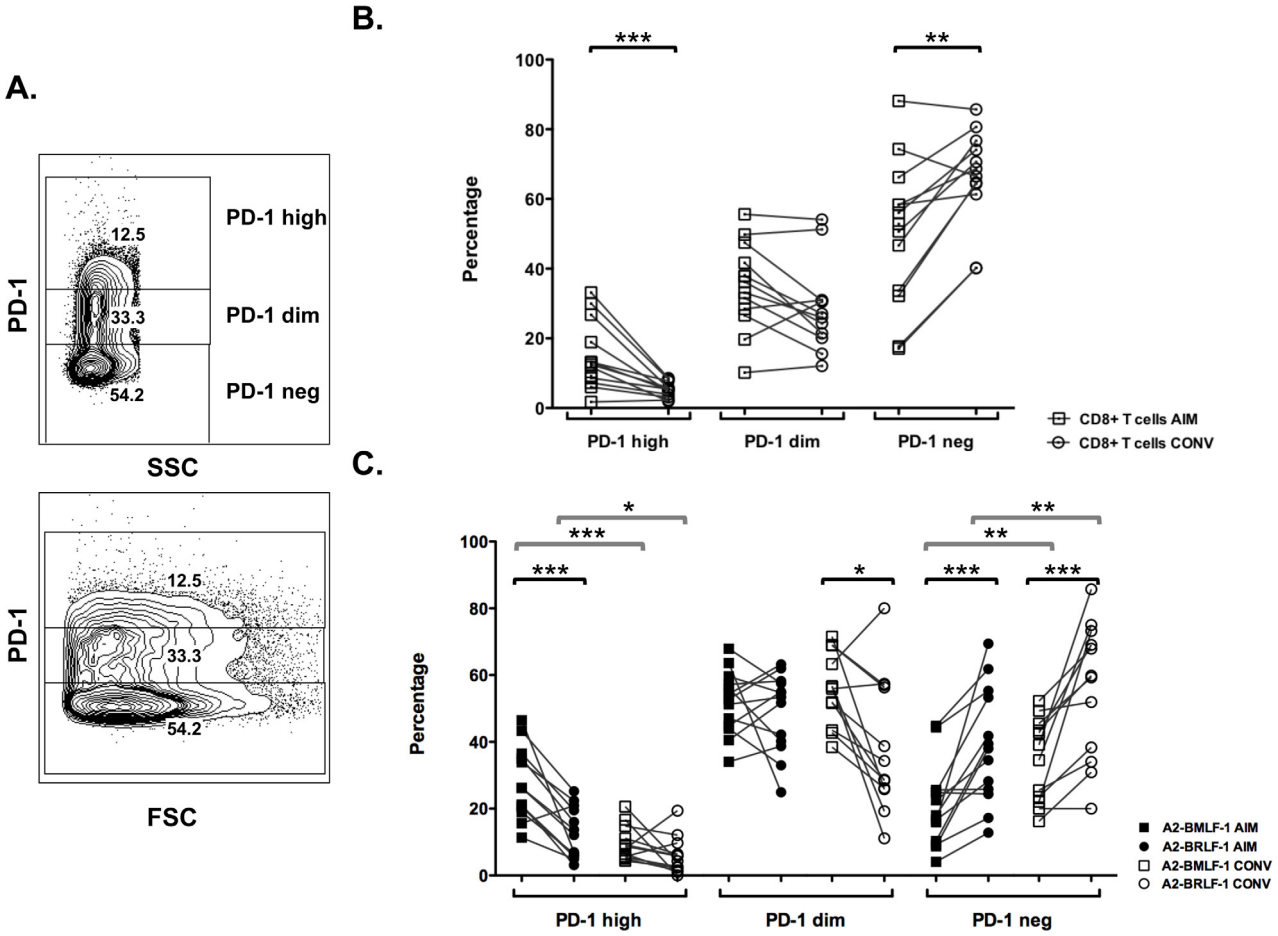
High level PD-1 expression on CD8+ T cells during AIM and convalescence. **A**. Representative CD8+ T cell plots of a sample obtained during AIM demonstrating the gating strategy to define PD-1 high, dim and negative populations. **B**. Percentages of PD-1 high, dim, or negative CD8+ T cells in AIM and convalescence. **C**. Percentages of PD-1 high, dim, or negative A2-BMLF-1 and A2-BRLF-1 CD8+ T cells in AIM and convalescence. Lines define paired results for each individual. Brackets indicate that differences between selected comparisons are significant (Wilcoxon signed rank test; *p<0.05, ** p<0.01, *** p<0.001).

Because high levels of expression of PD-1 are associated with loss of function or “exhaustion” [2], we also evaluated cell populations that were PD-1^high^, PD-1^dim^, or PD-1^negative^. Using a gating strategy based on a consistent contour “shoulder” observed in CD8+ T cell population plots of PD-1 against FSC or SSC (Figure 3A), we were able to analyze our data in a manner consistent with previous reports [14]. In total, CD8+ T cells expressed PD-1 at higher intensities during AIM (median 12.5; range 1.8 to 33.2) compared with convalescence (median 5.3; range 1.9 to 8.6; Figure 3B). These convalescent values are similar to those reported for HIV-1 infected individuals on effective antiretroviral therapy [14].

Among EBV-specific CD8+ T cells, only A2-BMLF-1 and A2-BRLF-1 CD8+ T cells were present in frequencies sufficient for analysis at this level of detail. High-level expression of PD-1 (PD-1^high^) was detected on a significantly greater percentage of EBV-specific CD8+ T cells during AIM than during convalescence (Figure 3C). For example, the frequencies of A2-BMLF-1 CD8+ T cells that were PD-1^high^ during AIM ranged from 11.3 to 46.5% (median 26.2%), and during convalescence ranged from 4.4 to 20.6% (median 8.4%). Thus the percentages of EBV epitope-specific CD8+ T cells with high-level PD-1 expression changed significantly between AIM and convalescence (Wilcoxon signed rank test, p<0.001 and 0.027 for A2-BMLF-1 and A2-BRLF-1 CD8+ T cells, respectively).

In summary, we have demonstrated higher frequencies of PD-1+ EBV-specific CD8+ T cells, as well as higher intensity of PD-1 expression during AIM compared with convalescence. These findings are consistent with the hypothesis that PD-1 expression is largely driven by antigenic stimulation. The differences in intensity of PD-1 expression (MFI and high level expression) are likely to be most biologically meaningful given prior studies demonstrating increased sensitivity to apoptosis and reduced proliferative capacity of CD8+ T cells with high intensity PD-1 staining [14], [34].

### Expression of PD-1 on CD8+ T Cells During AIM Correlates with Peripheral Blood EBV Load

We noted substantial variability in the frequency and intensity of PD-1 staining on CD8+ T cells between individual patients. In chronic lentiviral infections, the frequency and intensity of PD-1 expression on virus-specific CD8+ T cells directly correlates with plasma viral RNA copy number (a direct measure of viral replication) [16].

EBV replication is thought to occur primarily in oropharyngeal B cell blasts and epithelial cells; direct measurement of EBV replication at these sites is difficult. Peripheral blood EBV load (EBV DNA copy numbers in latently-infected memory B-cells) is easily measurable. Hadinoto et al [35] have demonstrated that high levels of EBV lytic replication during AIM results in very high levels of circulating latently-infected memory B cells; a marked reduction in peripheral blood EBV load is observed during convalescence as EBV-specific CD8+ T cells limit rounds of lytic viral replication and resultant seeding of the peripheral blood memory B cell pool.

**Figure 4:**
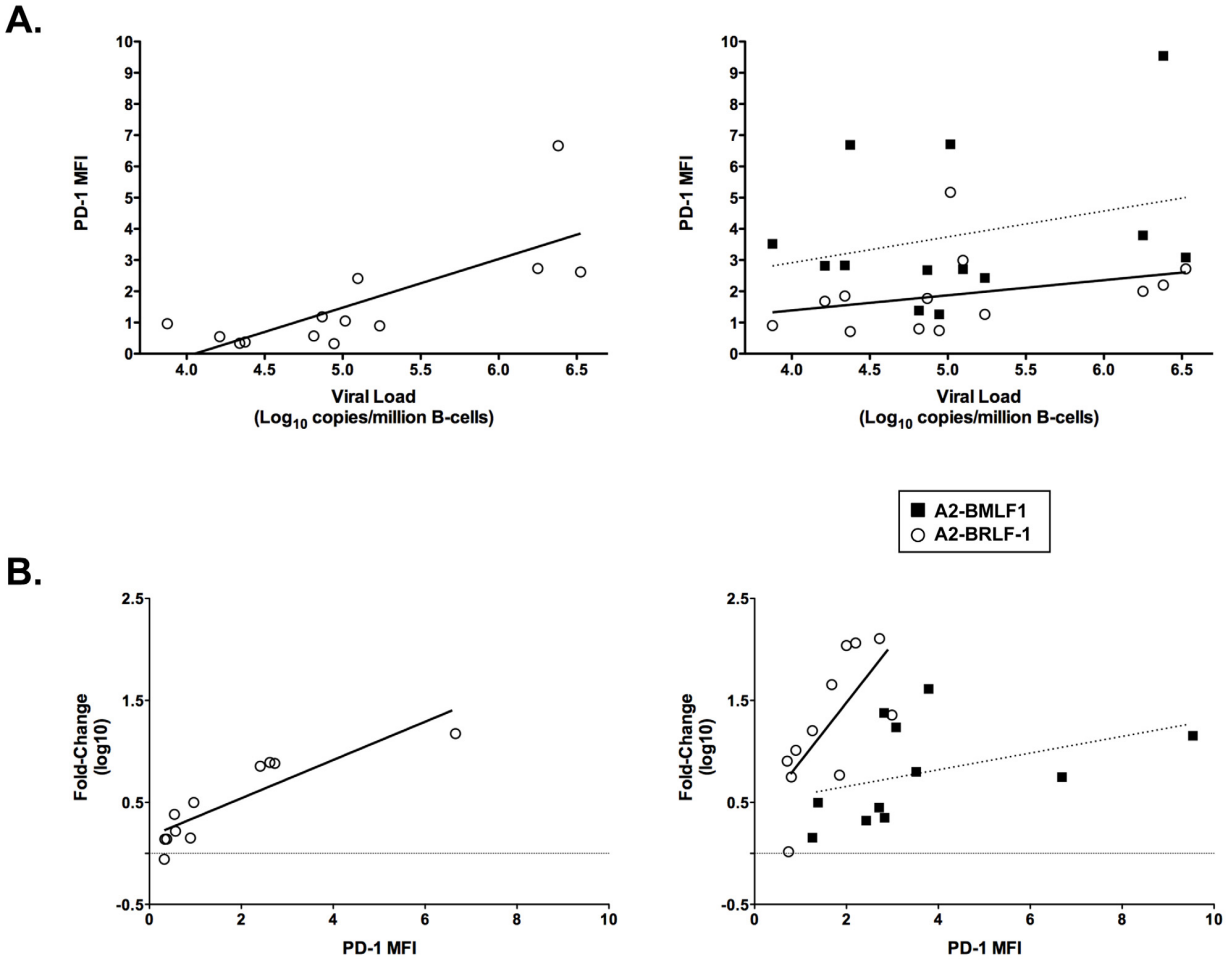
PD-1 expression on CD8+ T cells during AIM: correlations with viral load and contraction at convalescence. **A**. Left panel: Viral load (log transformed) and the corresponding PD-1 MFI of CD8+ T cells are plotted (Spearman r = 0.698; p = 0.008). Right panel: Viral load (log transformed) and the corresponding PD-1 MFI of A2-BMLF-1 (solid squares, dotted line) and A2-BRLF-1 cells (open circles, solid line) are plotted (Spearman r = 0.170; p = 0.578 and r = 0.560; p = 0.046, respectively). **B**. Left panel: PD-1 MFI of CD8+ T cells during AIM and the corresponding fold-change in CD8+ T cells (log-transformed) are plotted (Spearman r = 0.955; p<0.0001). The fold-change in CD8+ T cells is the ratio of the cell #/ml during AIM over the cell #/ml during convalescence. Right panel: PD-1 MFI of A2-BMLF-1 (solid squares, dotted line) and A2-BRLF-1 cells (open circles, solid line) during AIM and the corresponding fold-change in each of these subsets (log-transformed) are plotted (Spearman r = 0.646; p = 0.037, and r = 0.764; p = 0.009, respectively). Lines of best fit are shown for each combination of variables.

In samples obtained during AIM (a period of active EBV replication and seeding of the peripheral blood memory B cell pool), there was a significant positive correlation between the viral load (EBV DNA copies per million B cells) and the MFI of CD8+ T cells expressing PD-1 (Figure 4A, left panel; Spearman correlation: r = 0. 698; p = 0.008). The CD8+ T cell PD-1 percentage was also significantly correlated with EBV load (data not shown; Spearman correlation: r = 0.747; p = 0.003). PD-1 expression on both A2-BMLF-1 and A2-BRLF-1 CD8+ T cells showed a weaker positive correlation with viral load during AIM, reaching statistical significance only for A2-BRLF-1 CD8+ T cells (Figure 4A, right panel; A2-BRLF-1 PD-1 MFI and viral load; Spearman correlation: r = 0.560; p = 0.046). PD-1 expression on CD8+ T cells did not correlate with viral load in samples obtained during convalescence.

### Expression of PD-1 on CD8+ T Cells During AIM Correlates with Contraction in Convalescence

It has been reported that EBV-specific responses that are differentially “culled” may have different levels of PD-1 expression; higher PD-1 expression was detected on certain epitope-specific CD8+ T cells at time points just prior to the disappearance of these CD8+ T cells from the peripheral circulation [31]. In animal models of uncontrolled infection, PD-1 expression decreased on epitope-specific CD8+ T cells as they decreased in frequency in the context of emerging viral escape mutations [16], [34], [36]. We, therefore, examined the relationship between PD-1 expression on EBV epitope-specific CD8+ T cells during AIM and the degree to which these cell populations contracted between AIM and convalescence.

We observed a significant positive correlation between the intensity of PD-1 expression and the fold-change (decrease) in the absolute number of circulating CD8+ T cells between AIM and convalescence (Figure 4B, left panel; MFI vs. fold-change; Spearman r = 0.955; p<0.0001). This relationship was also observed for the percentage of PD-1 positive CD8+ T cells; Spearman r = 0.891; p = 0.0005; data not shown). The intensity of PD-1 expression on both A2-BMLF-1 and A2-BRLF-1 CD8+ T cells also showed a significant positive correlation with the degree of contraction between AIM and convalescence (Figure 4B, right panel; Spearman correlations and p-values, respectively: r = 0.646, p = 0.037 and r = 0.764, p = 0.009). This relationship was also observed for the percentage of PD-1 positive cells in each of these subsets, but reached significance only for the A2-BRLF-1 CD8+ T cells (data not shown; Spearman correlation: r = 0.665; p = 0.031).

These data are consistent with antigen driven expansion and upregulation of PD-1 on virus-specific CD8+ T cells during AIM. However, PD-1 expression on A2-BRLF-1 CD8+ T cells was more strongly associated with viral load and the degree of contraction than was PD-1 expression on A2-BMLF-1 CD8+ T cells.

### Consistent Differences in PD-1 Expression are Observed on EBV Epitope-specific CD8+ T Cells

**Figure 5:**
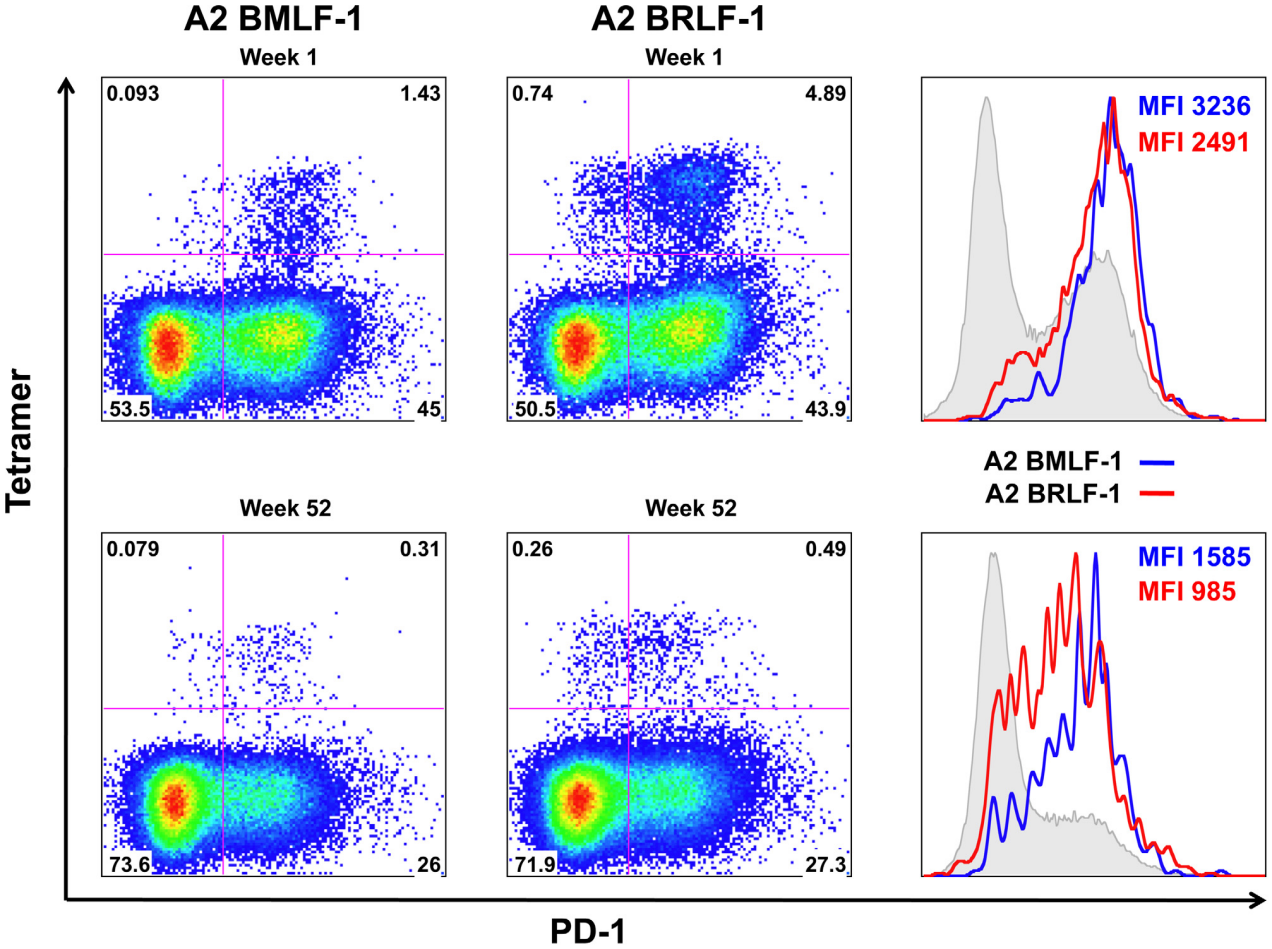
Differential PD-1 expression on A2-BMLF-1 and A2-BRLF-1 CD8+ T cells at AIM and convalescence. PD-1 expression on A2-BMLF-1 and A2-BRLF-1 CD8+ T cells during AIM (upper panels) and convalescence (lower panels). Representative dot plots demonstrate differences in PD-1 percent positivity, and histograms showing differences in distribution and PD-1 MFI of these EBV-specific CD8+ T cells. Histograms overlay the two subsets on a background of the total CD8+ T cell population.

In our analyses, we consistently observed lower frequencies and intensities of PD-1 expression on A2-BRLF-1 CD8+ T cells compared with A2-BMLF-1 CD8+ T cells. A representative example of this is shown in Figure 5. In this example, 94% of A2-BMLF-1 and 87% of A2-BRLF-1CD8+ T cells during AIM expressed PD-1. At this time point, the MFI of PD-1 expression for A2-BMLF-1 and A2-BRLF-1 were 3,236 and 2,491, respectively. At week 52 (convalescence), 80% of A2-BMLF-1 and 65% of A2-BRLF-1 CD8+ T cells expressed PD-1, and PD-1 MFI were 1,585 and 985, respectively. Overall, the percentages of A2-BMLF-1 CD8+ T cells that expressed PD-1 were significantly greater than the percentages of A2-BRLF-1 CD8+ T cells, both during AIM and convalescence (Figure 2A; Wilcoxon signed rank test, p<0.01 for both comparisons). In addition, we observed higher intensity (MFI) of PD-1 staining in A2-BMLF-1 CD8+ T cells compared with A2-BRLF-1 CD8+ T cells (Figure 2B; Wilcoxon signed rank test, p<0.01 for both comparisons). Finally, significantly higher frequencies of A2-BMLF-1 CD8+ T cells expressed PD-1 at high levels (PD-1^high^) during AIM compared with A2-BRLF-1 CD8+ T cells (Figure 3C; Wilcoxon signed rank test, p<0.001). During both AIM and convalescence, we observed a lower frequency of PD-1^negative^ A2-BMLF-1 CD8+ T cells compared with A2-BRLF-1 CD8+ T cells (Figure 3C; Wilcoxon signed rank test, p<0.001 for both comparisons).

Again, this analysis included measures of the intensity of PD-1 expression (MFI and PD-1^high^ percentage) on virus-specific CD8+ T cells because they are considered to be more biologically meaningful. Given the higher degree of contraction of A2-BRLF-1 CD8+ T cells (48.7-fold decrease in mean values) following AIM, and prior reports that PD-1 expression is significantly correlated with contraction, it was surprising to find that the intensity of PD-1 expression was lower than that of A2-BMLF-1 CD8+ T cells both in AIM and convalescence. Data presented in Figure 4B (right panel) underscores the observation of consistently higher levels of PD-1 expression on A2-BMLF-1 CD8+ T cells compared with A2-BRLF-1 CD8+ T cells. It is also evident that there may be a significant correlation between PD-1 expression and degree of contraction for a particular subset of virus-specific CD8+ T cells, however this relationship may differ substantially for another subset.

We (Catalina, et al 2001), and others have previously shown that EBV latent epitope-specific CD8+ T cells undergo lower levels of expansion and contraction than lytic epitope-specific CD8+ T cells [24], [29]. A robust comparison of PD-1 expression on lytic and latent antigen-specific CD8+ T cells was not feasible due to the markedly lower frequencies of detectable latent antigen-specific CD8+ T cells. However, in samples where latent epitope specific CD8+ T cells were also detected in sufficient numbers (e.g., A2-EBNA-3c and B7-EBNA-3a CD8+ T cells; Figure 2), PD-1 expression levels generally exceeded those measured on A2-BRLF1 CD8+ T cells. These data suggest that PD-1 expression may not be the sole factor influencing contraction of a CD8+ T cell response in convalescence.

### Differences in PD-1 Expression on EBV-specific CD8+ T Cells are not Related to Measurable Differences in Differentiation State or Activation

PD-1 expression on virus-specific CD8+ T cells has been regarded as a sensitive marker of antigenic stimulation [16]. The differences in PD-1 expression on A2-BMLF-1 and A2-BRLF-1 CD8+ T cells could be consistent with quantitative or qualitative differences in antigen stimulation. If this were the case, these cells would be expected to display differences in other markers of differentiation or activation. We studied a subset of our population (n = 5) to determine whether there were any identifiable differences in differentiation between A2-BMLF-1 and A2-BRLF-1 CD8+ T cells. We found that the major differentiation states defined by CD27, CD28 and CD127 were similarly represented in these two EBV-specific CD8+ T cell subsets (data not shown). The highest PD-1 levels were observed on effector memory cells in early/intermediate stages of differentiation (CD27+28+127−) both in AIM and convalescence.

High frequencies (92 to 100%) of EBV epitope-specific CD8+ T cells expressed HLA-DR during AIM. Fewer A2-BMLF-1 (16 to 48%, median 28%) and A2-BRLF-1 (6 to 41%; median 22%) CD8+ T cells expressed HLA-DR during convalescence. HLA-DR expression did not correlate with PD-1 expression during AIM or convalescence (data not shown).

### PD-1 Expression on EBV-specific CD8+ T Cells Does Not Correlate with CD127 Expression

Previous reports have related CD127 (IL-7R) expression with the expansion and contraction of EBV-specific CD8+ T cells in AIM and convalescence [26]. Sauce et al [31] have recently reported a gradual increase in CD127, and a transient high intensity upregulation of PD-1 expression on EBV epitope-specific CD8+ T cells prior to their disappearance from the circulation. Our analysis did not detect evidence of higher PD-1 expression on a more rapidly contracting population of EBV-specific CD8+ T cells (the A2-BRLF-1 CD8+ T cells), so we examined CD127 expression in greater detail.

**Figure 6:**
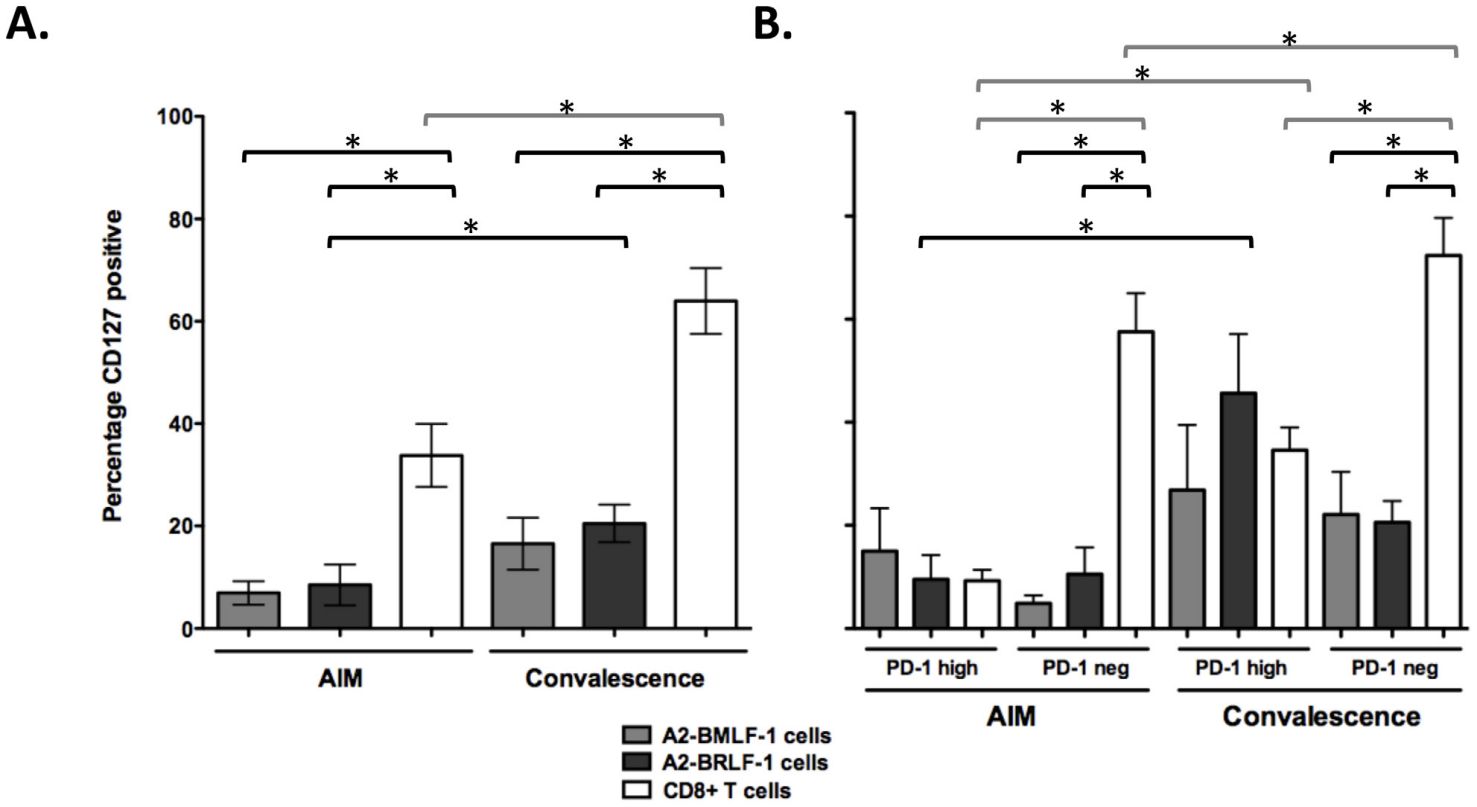
Analysis of CD127 and PD-1 expression on CD8+ T cells. **A**. Percentages of A2-BMLF-1, A2-BRLF-1, and total CD8+ T cells that are CD127+ in AIM and convalescence (mean and SEM). **B**. Percentages of A2-BMLF-1, A2-BRLF-1, and total CD8+ T cells subsets that are PD-1 high or PD-1 negative that are also CD127+ (in AIM and convalescence). Brackets indicate paired comparisons that differ significantly, Wilcoxon signed rank test, *p<0.05.

CD127 expression was examined from AIM through convalescence in seven individuals. The percentages of A2-BMLF-1 and A2-BRLF-1 CD8+ T cells that expressed CD127 ranged from 0.7 to 18.9% (median 5.8%) and 0.4 to 31.6% (median 6.1%) during AIM, respectively. The frequencies of CD127+ virus-specific CD8+ T cells trended upward in convalescence but reached significance only for A2-BRLF-1 CD8+ T cells (Figure 6A; Wilcoxon signed rank test, p = 0.047).

Within PD-1^high^ subpopulations, EBV-specific CD8+ T cells expressed CD127 at low frequencies that did not differ from the total CD8+ T cell population either during AIM or convalescence (Figure 6B). Within PD-1^negative^ subpopulations, EBV-specific CD8+ T cells expressed CD127 at low frequencies that did not differ from PD-1^high^ subpopulations at either time point.

### Reduced Proliferative Capacity of EBV Epitope-specific CD8+ T Cells in AIM Regardless of PD-1 Expression Levels

**Figure 7:**
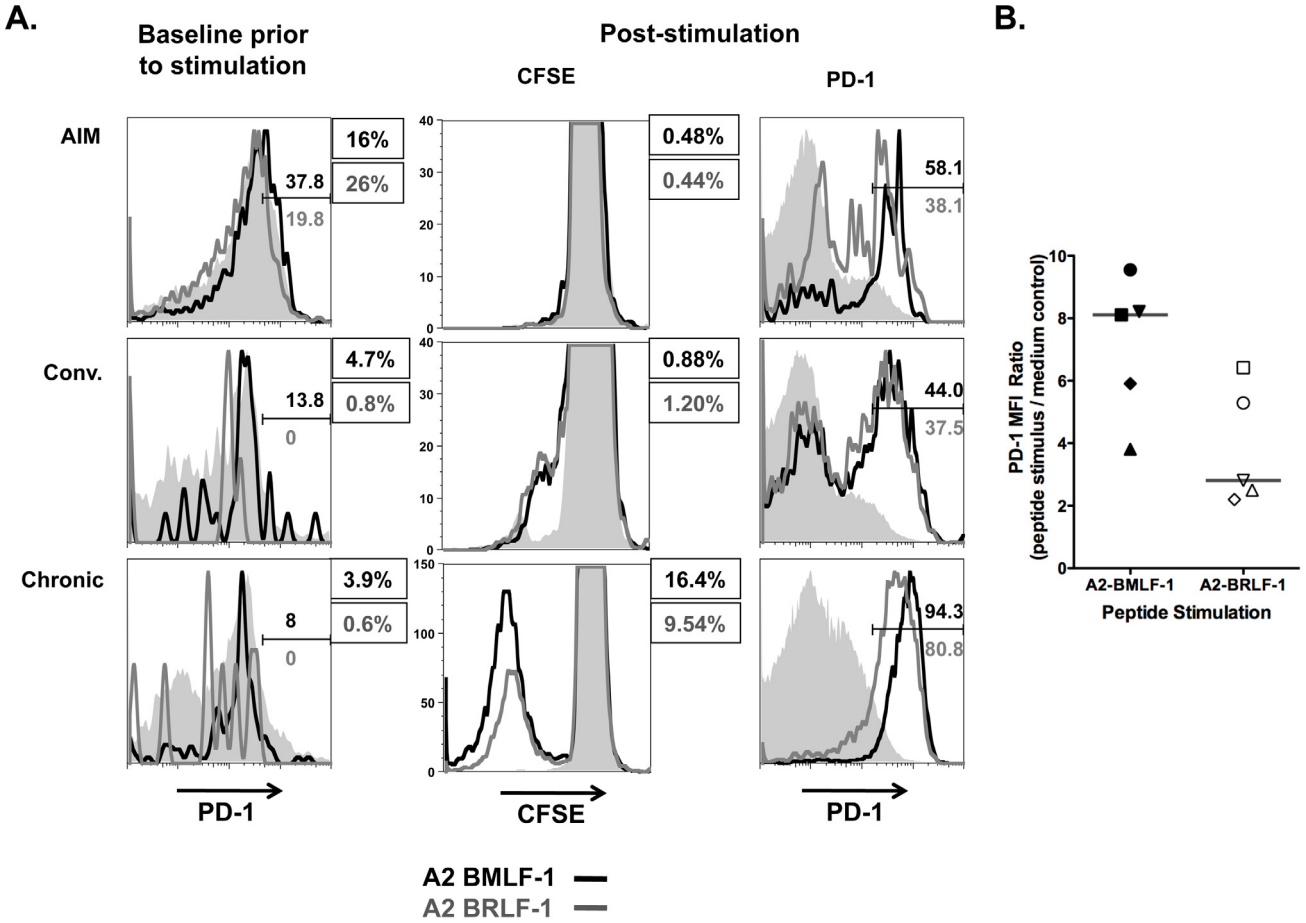
Proliferation and PD-1 expression on CD8+ T cells responding to peptide stimulation. **A**. Three representative examples of proliferation upon stimulation with HLA-A*0201-restricted BMLF-1 and BRLF-1 peptides. Upper and middle rows: PBMC from an individual at presentation with AIM and in convalescence; lower row: PBMC from an individual with chronic infection. Left panels (baseline prior to peptide stimulation) show PD-1 expression of the indicated tetramer positive cells (A2 BMLF-1 black line; A2 BRLF-1 grey line) superimposed on that of CD8+ T cells (grey solid). Middle panels show CD8+ T cells that proliferate (CFSE dilution) in response to the indicated peptides (A2 BMLF-1 black line; A2 BRLF-1 grey line) superimposed on unstimulated controls (grey solid). Right panels show the upregulation of PD-1 on CD8+ T cells responding to the indicated peptides superimposed on unstimulated CD8+ T cells. Marked regions on histograms depicting PD-1 expression indicate high levels of expression with corresponding percentages of A2 BMLF-1 and A2-BRLF-1 CD8+ T cells. Numbers in text boxes are the percentages of CD8+ T cells that are positive for each tetramer at baseline (left panels), or responding to peptide post-stimulation (CFSE low; middle panels). **B**. Upregulation of PD-1 on cells responding to stimulation with A2-BMLF-1 and A2-BRLF-1 peptides. Results shown are a mixture of chronic and convalescent samples in three separate assays. To demonstrate the extent of PD-1 upregulation observed, PD-1 MFI is expressed as a ratio of the MFI of cells that responded to peptide, relative to the MFI in control assays without peptide. Median PD-1 MFI ratio values for A2-BMLF-1 and A2-BRLF-1 were 8.1 and 2.8, respectively (Wilcoxon signed rank test; p = 0.06). Solid and open symbols demonstrate paired values for A2-BMLF-1 and A2-BRLF-1 for each sample.

High intensity expression of PD-1 on CD8+ T cells has correlated most consistently with poor proliferation and sensitivity to apoptosis [34]. In samples from individuals with AIM, we consistently observed dramatic EBV epitope-specific CD8+ cell loss in tissue culture when compared with samples obtained in convalescence. This cell loss occurred spontaneously (even without stimulation) and was associated with a profound lack of response to EBV peptide stimulation (Figure 7A, top panels). In AIM, neither A2-BMLF-1 nor A2 BRLF-1 CD8+ cells proliferated in response to peptide stimulation. The degree to which these EBV epitope-specific cells were lost over the course of a 6-day assay was greater than would be predicted from the percentage of cells expressing high PD-1 levels. In the representative AIM example shown in Figure 7A, the frequencies of A2-BMLF-1 and A2-BRLF-1 CD8+ T cells expressing high levels of PD-1 at baseline prior to stimulation were 37.8% and 19.8%, respectively. For each assay condition it was estimated that 217,500 A2-BMLF-1 CD8+ T cells and 322,500 A2-BRLF-1 CD8+ T cells were present initially. After 6 days with peptide stimulation, there were fewer than 200 cells that responded specifically to the BMLF-1 or the BRLF-1 peptide (by CFSE dilution). These decreases were also documented by similar reductions in tetramer positive cells (data not shown). From these data, it was apparent that both PD-1 positive and negative virus-specific cells failed to proliferate. The lack of responsiveness to stimulation and cell loss in AIM were virus specific; proliferative responses to Candida antigen were preserved in AIM and convalescence (data not shown).

In samples obtained during convalescence, A2-BMLF-1 and A2-BRLF-1 CD8+ T cells proliferated to a similar extent with peptide stimulation. The median fold-change in frequency of peptide responsive cells relative to baseline frequency of tetramer positive cells for A2-BMLF-1 CD8+ T cells was 2.1 (range 0.4 to 23.3), and for A2-BRLF-1 CD8+ T cells was 4.3 (range 1.2 to 23.8). There was no evidence of impaired proliferation with the A2-BMLF-1 peptide compared to the A2-BRLF-1 peptide; indeed in some assays, A2-BMLF-1 proliferation was more pronounced despite higher expression of PD-1 on these cells (Figure 7A, chronic infection).

The addition of antibody that blocked PD-1/PD-L1 interactions to assays on samples from AIM patients did not reverse the observed defects in proliferation. In contrast, samples from convalescent individuals demonstrated enhanced proliferative responses to BMLF-1 and BRLF-1 peptides (1.3–2.1-fold, mean 1.8-fold) in the presence of antibody to block PD-1/PD-L1 interactions (data not shown).

PD-1 expression was upregulated on cells responding to peptide stimulation (Figure 7A). In a series of assays, the PD-1 MFI of peptide stimulated CD8+ T cells ranged from approximately 2 to 10 times that of medium control. Interestingly, the pattern of PD-1 expression following stimulation was similar to that observed *ex vivo*; CD8+ T cells responding to A2-BRLF-1 peptide had lower intensity PD-1 expression than those responding to the A2-BMLF-1 peptide (Figure 7B).

### PD-1 Expression on V-beta Subsets of EBV Lytic Antigen Epitope Specific CD8+ T Cells Varies Significantly

The observed differences in PD-1 expression on A2-BMLF-1 and A2-BRLF-1 CD8+ T cells raised the possibility that PD-1 expression may be influenced significantly by factors other than antigen presentation. We hypothesized that differences in the effectiveness of TCR engagement and signaling would also influence PD-1 expression. If the main determinant of PD-1 expression level were antigenic stimulation, then different clonotypes within A2-BMLF-1 or A2-BRLF-1 populations would be expected to have similar levels of PD-1 expression (assuming that all subsets of cells with a given epitope specificity are presented with a similar level of antigen in a similar context and time frame). If, however, significant variability in PD-1 expression were to exist among clonotypes with identical epitope specificity, then factors specific to particular clonotypes of responding cells would be implicated as determinants in addition to the level of antigen presentation.

**Figure 8:**
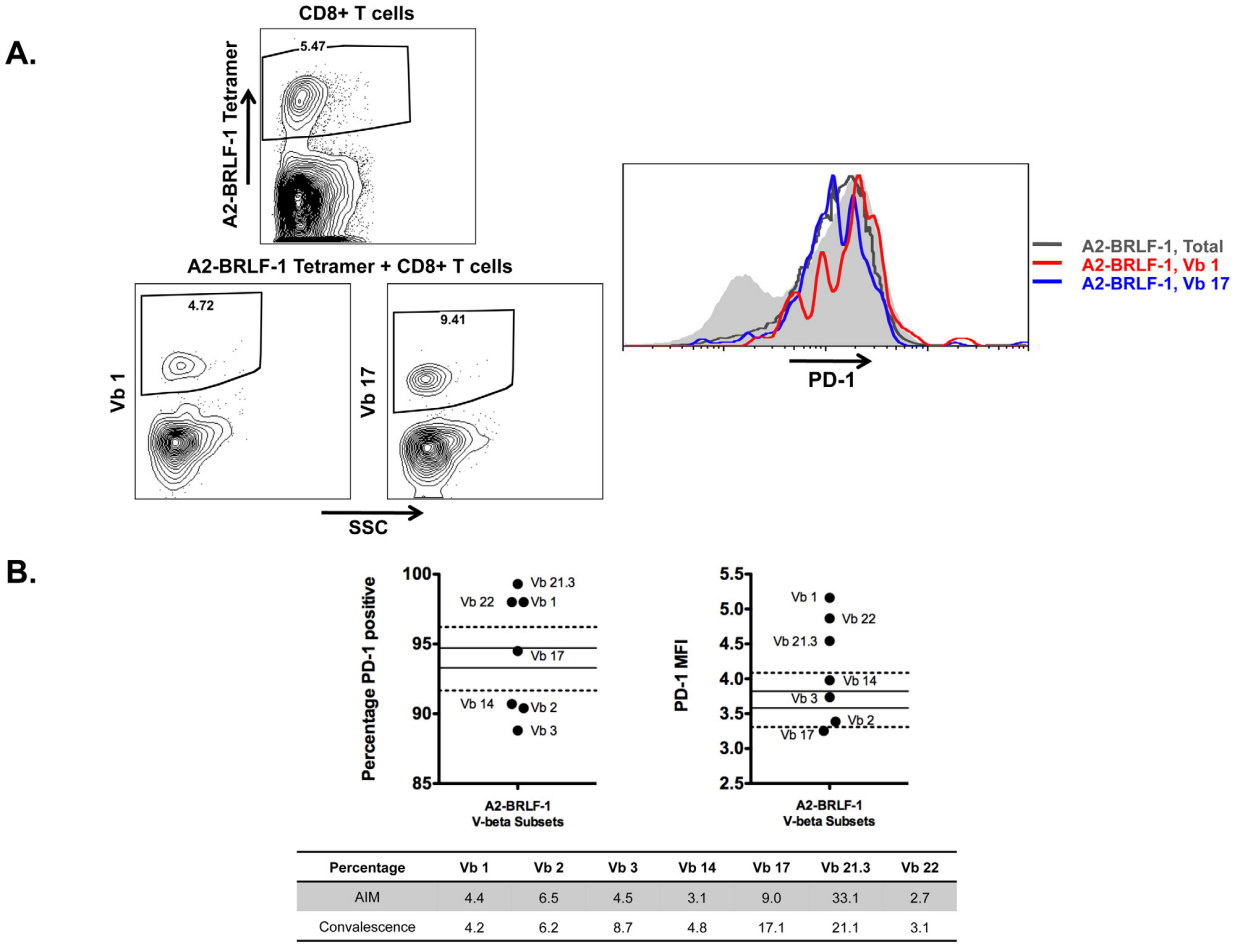
PD-1 expression on V-beta subsets of A2-BRLF-1 CD8+ T cells in AIM. **A**. Representative data from one individual. Left panels show dot plots of regions defining A2 BRLF-1 CD8+ T cells, and Vb 1 and Vb 17 subsets. Right panel is a histogram showing the PD-1 expression of all A2 BRLF-1 CD8+ T cells (grey line), and the A2 BRLF-1 Vb 1 (red line), and A2 BRLF-1 Vb 17 (blue line) subsets superimposed on the PD-1 expression of the total CD8+ T cell population (grey solid). **B**. PD-1 expression on all V-beta subsets of A2 BRLF-1 CD8+ T cells studied in this individual. Percentage and MFI are shown in left and right panels, respectively. The corresponding measurements of the total tetramer positive population were used to calculate mean and 95% confidence intervals shown (solid horizontal lines). Also shown is the range of values defined by the mean +/− 3*SD (dotted horizontal lines). PD-1 percentage and MFI values outside the more stringent ranges are considered significantly different from the total tetramer positive population. The table inset shows the percentages of the V-beta subsets of A2-BRLF-1 CD8+ T cells in AIM and convalescence for this individual.

To address this question, we examined PD-1 expression on V-beta subsets of A2-BMLF-1 and A2-BRLF-1 CD8+ T cells from three individuals with AIM. Subsets with sufficient numbers of analyzable events (mean 129; range 24 to 702) were selected. These V-beta subsets were compared with the total tetramer positive population (representative gating and PD-1 expression shown in Figure 8A). Multiple measurements of PD-1 expression on Tetramer positive cells in a given assay enabled calculation of mean and standard deviation (SD). For all three individuals, we observed clear examples of V-beta subpopulations that had PD-1 expression significantly higher or lower than the range defined by mean +/− 3*SD (representative data shown in Figure 8B). At early visits, these differences did not appear to predict loss or enrichment of particular V-beta subsets at subsequent visits (Figure 8B). For example, Vb 21.3 had high PD-1 expression, yet persisted at a relatively high percentage (21.1%) in convalescence. Nor did PD-1 expression correlate with the frequency of a particular V-beta subset; high PD-1 expression was found on subsets that were present at persistently low or high frequencies (e.g. Vb 22 frequencies ranged between 2.7 and 3.1% and Vb 21.3 frequencies ranged between 33.1 and 22.1% despite both expressing high frequencies and intensities of PD-1).

## Discussion

Consistent with prior reports, our analyses suggest that PD-1 expression is driven in large part, but not solely, by viral antigen expression. Moreover, PD-1 expression alone does not reflect the functional capacity of CD8+ T cells. Factors other than PD-1 appear to participate in: 1) modulating EBV-specific CD8+ T cell proliferative capacity during both AIM and convalescence, 2) determining states of differentiation and activation, 3) and the degree of contraction of EBV-specific CD8+ T cells in convalescence. These observations serve to refine and extend our understanding of the complex network of factors that govern immune responses and tolerance. To our knowledge, this is the first demonstration that PD-1 expression on virus-specific CD8+ T cells is influenced significantly by factors intrinsic to the responding cell that segregate with epitope specificity and V-beta usage. While these factors have yet to be defined, these observations would be consistent with variable TCR binding and signaling characteristics that may play a significant role in shaping effective immune responses. Price et al have shown that immunodominant clonotypes of EBV and CMV specific CD8+ T cells tend to have greater avidity for their cognate antigen [37]. While we have not shown a relationship between PD-1 and the frequency of V-beta usage (i.e. immunodominance), it remains possible that avidity relates to PD-1 expression.

Previous studies have emphasized a relationship between high levels of antigenic stimulation and high PD-1 expression levels on virus-specific CD8+ T cells in chronic viral infections. In a murine model of LCMV infection, graded levels of PD-1 expression on virus-specific CD8+ T cells correlated with viral clearance rates at day 30 after infection [38]. The highest levels of PD-1 expression were found on exhausted virus-specific CD8+ T cells in animals with chronically elevated LCMV titers. Whereas PD-1 expression on virus-specific CD8+ T cells in untreated HIV-1 (or SIV in animal models of AIDS) infection is high and is directly related to viral replication [8], [13], [34], PD-1 expression on CMV-specific CD8+ T cells is reportedly lower; presumably due to intermittent or lower levels of viral antigen expression. Relatively high levels of PD-1 expression on EBV-specific CD8+ T cells from chronically infected individuals have been reported by a number of groups [8], [13]. Most studies that have compared PD-1 expression on EBV vs. HIV or CMV specific CD8+ T cells have focused on A2-BMLF-1 CD8+ T cells [8], [13]. Our results are in agreement with these previous reports, however, we have also demonstrated consistent and significant differences in PD-1 expression among different epitope specific CD8+ T cell subsets.

The concept that circulating virus-specific CD8+ T cells with different epitope specificities may display consistent differences in PD-1 expression has precedent in the literature. Using a broad panel of pMHCI tetramers to characterize HIV-specific CD8+ T cell responses in infected individuals, Day and colleagues observed lower levels of PD-1 expression on certain epitope-specific CD8+ T cells among a majority that expressed PD-1 at high levels [8]. A dramatic difference in PD-1 expression on virus-specific CD8+ T cells with different epitope specificities has also been reported in an individual with resolved HCV infection [10]. Our data demonstrated consistent differences in PD-1 expression on A2-BMLF-1 and A2-BRLF-1 CD8+ T cells over the course of infection from AIM to convalescence, and between different individuals. This suggests a mechanism that relates to differences in TCR signaling that are shared by HLA-A*0201 positive individuals responding to these epitopes. Possible mechanisms include: 1) differences in the stability of the peptide/MHC-1 association, or 2) a common limitation on TCR signaling (perhaps defined by the avidity of the TCR for the pMHCI complex). Our data show that exposure of epitope-specific CD8+ T cells to high (presumably saturating) peptide concentrations result in different degrees of PD-1 upregulation, consistent with PD-1 expression ex vivo. This is unlikely to be a function of limiting concentrations of pMHCI complexes on the antigen presenting cells. MHC-peptide binding algorithms actually predict that the A2-BRLF-1 epitope has a longer half-life of dissociation from HLA-A*0201 than the A2-BMLF-1 epitope. If antigen presentation were the most critical factor, the less stable pMHCI complex might be expected to be associated with lower PD-1 expression. Therefore, it is more likely that there is a determinant of PD-1 upregulation specific to the cells responding to these particular epitopes. The observation that PD-1 expression may vary significantly in different V-beta subsets that recognize the same epitope suggests that V-beta usage itself may influence PD-1 expression. Further studies are necessary to better define determinants of PD-1 expression on virus-specific CD8+ T cells and their functional consequences.

Given our current understanding of EBV replication, it is likely that different EBV lytic antigens are presented to CD8+ T cells in a similar microenvironment, and in a similar time frame. The differences in PD-1 expression on A2-BMLF-1 and A2-BRLF-1 CD8+ T cells, despite highly similar differentiation phenotypes, suggests a dissociation between signaling pathways that determine differentiation states and PD-1 expression. We have examined the relationship between PD-1 expression and CD127 in detail because a recent report indicates that CD127 expression is tightly linked with proliferative capacity, a function that is lost early as PD-1 is upregulated [7]. Furthermore, upregulation of both PD-1 and CD127 has been reported to precede disappearance of an EBV-specific CD8+ T cell from the circulation [31]. We found that few CD8+ T cells expressing high levels of PD-1 also expressed CD127 in AIM, but this relationship was not exclusive; higher frequencies of CD127+, PD-1+ cells were seen in convalescence. As reported by Salisch et al, PD-1 expression on virus-specific CD8+ T cells is a sensitive indicator of lentiviral replication [16]. Kasprowicz et al have also suggested that PD-1 staining is compatible with it being an activation marker [10]. Our analyses are consistent with these interpretations. While PD-1 levels correlated with the contraction of CD8+ T cells, we did not find a simple association between higher PD-1 levels on EBV-specific CD8+ T cells and the degree of contraction from AIM to convalescence. Within a particular epitope specific subset (e.g. A2-BRLF-1 CD8+ T cells), there can be a significant correlation, however between subsets, similar levels of contraction occur with significantly different PD-1 expression (Figure 4B, right panel). This suggests that PD-1 may be necessary, but not sufficient in this process.

Sauce et al [31] have described transient increases in PD-1 expression on epitope-specific CD8+ T cells that are associated with an “accelerated” expression of CD127 and subsequent disappearance of that epitope-specific response from the circulation. Consistent differences in PD-1 staining intensity were not reported in their analysis of A2-BMLF-1 (labeled “GLC”) than A2-BRLF-1 (labeled “YVL”) CD8+ T cells in acutely infected individuals at all time points to convalescence. However, careful inspection of the data presented actually demonstrates higher PD-1 expression on A2-BMLF-1 than A2-BRLF-1 CD8+ T cells in three of four individuals at multiple time points, and in the fourth subject during acute infection. Differences in PD-1 expression may have been less evident due to the binding characteristics of the anti-PD-1 antibody used. Indeed, we had collected additional earlier data using a different anti-PD-1 antibody that failed to clearly distinguish positive and negative populations, yet still demonstrated these differences in PD-1 expression at all time points (data not shown). Using another antibody (Medarex), we observed PD-1 expression profiles with clearly defined bimodal distributions (data reported here in Figures 3A, 5, 7A, 8A). The ability to distinguish differences in intensity of PD-1 expression, particularly PD-1^high^ expression, has been previously shown to be critical for describing meaningful biological differences.

The functional impact of PD-1 upregulation on EBV-specific CD8+ T cells during AIM is unclear. We found virtually no proliferation to peptide stimulation during AIM, and no enhancement of proliferation in the presence of antibody blocking the PD-1/PD-L1 interaction. While PD-1 upregulation may contribute to loss of responsiveness, it certainly cannot explain it in entirety. Our findings are consistent with other reports that PD-1 expression alone does not explain loss of proliferative capacity or sensitivity to apoptosis [12], [39]. In convalescence, the inhibitory activity associated with PD-1 is more evident; enhanced proliferative responses are observed in the presence of anti-PD-L1. Our observations thus highlight the complexities of PD-1 expression and its functional correlates; in some settings (e.g. chronic LCMV infection in mice), its expression is consistent with cellular exhaustion [6], while in others (e.g. acute hepatitis C infection), it appears primarily to be a marker of activation [10]. Blackburn et al have shown that there may be multiple inhibitory molecules that contribute to an “exhausted” phenotype [38], and that PD-1/PD-L1 blockade primarily rescues proliferation in less terminally differentiated cells [39]. We have now shown that these complexities must be viewed through a lens that accounts not only for antigen expression, but also epitope specificity and V-beta usage.

In aggregate, studies indicate that PD-1 expression and signaling play an important role in limiting pathogenic consequences of chronic virus infection. Efforts are underway to determine if disrupting the interaction of PD-1 with its ligands will have a beneficial effect in chronic viral infections in which cellular immunity fails to provide control. We have shown that PD-1 expression is induced by antigenic stimulation, but is dependent in part on intrinsic properties of the responding cell. Consistent differences, and significant variability in PD-1 expression observed in two lytic epitope specific CD8+ T cell subsets, and subpopulations defined by V-beta usage point to properties that are likely linked to TCR signaling, TCR avidity and recruitment of molecules involved in signal transduction. Properties of TCR-pMHCI interactions that influence expression of PD-1 are likely to inform the future designs of vaccines and immunotherapeutics.
